# A historical literature review of coronary microvascular obstruction and intra-myocardial hemorrhage as functional/structural phenomena

**DOI:** 10.7555/JBR.37.20230021

**Published:** 2023-07-28

**Authors:** Leonid N. Maslov, Natalia V. Naryzhnaya, Sergey V. Popov, Alexandr V. Mukhomedzyanov, Ivan A. Derkachev, Boris K. Kurbatov, Andrey V. Krylatov, Feng Fu, Jianming Pei, Vyacheslav V. Ryabov, Evgenii V. Vyshlov, Svetlana V. Gusakova, Alla A. Boshchenko, Akpay Sarybaev

**Affiliations:** 1 Laboratory of Experimental Cardiology, Cardiology Research Institute, Tomsk National Research Medical Center of the Russian Academy of Sciences, Tomsk, Tomsk Region 634012, Russia; 2 Department of Physiology and Pathophysiology, National Key Discipline of Cell Biology, School of Basic Medicine, the Fourth Military Medical University, Xi'an, Shaanxi 710032, China; 3 Siberian State Medical University, Tomsk, Tomsk Region 634050, Russia; 4 National Center of Cardiology and Internal Medicine, Bishkek 720040, Kyrgyzstan

**Keywords:** heart, ischemia, reperfusion, microvascular obstruction, intra-myocardial hemorrhage

## Abstract

The analysis of experimental data demonstrates that platelets and neutrophils are involved in the no-reflow phenomenon, also known as microvascular obstruction (MVO). However, studies performed in the isolated perfused hearts subjected to ischemia/reperfusion (I/R) do not suggest the involvement of microembolization and microthrombi in this phenomenon. The intracoronary administration of alteplase has been found to have no effect on the occurrence of MVO in patients with acute myocardial infarction. Consequently, the major events preceding the appearance of MVO in coronary arteries are independent of microthrombi, platelets, and neutrophils. Endothelial cells appear to be the target where ischemia can disrupt the endothelium-dependent vasodilation of coronary arteries. However, reperfusion triggers more pronounced damage, possibly mediated by pyroptosis. MVO and intra-myocardial hemorrhage contribute to the adverse post-infarction myocardial remodeling. Therefore, pharmacological agents used to treat MVO should prevent endothelial injury and induce relaxation of smooth muscles. Ischemic conditioning protocols have been shown to prevent MVO, with L-type Ca^2+^ channel blockers appearing the most effective in treating MVO.

## Introduction

In 1966, Krug *et al* could not find the complete restoration of coronary blood flow (CBF) in cats after 60 min of coronary artery occlusion (CAO)^[1]^. In 1967, Majno *et al* reported the absence of the complete restoration of blood flow after rabbit brain ischemia (15 min)^[2]^. Investigators called this phenomenon "no-reflow"^[2]^. The no-reflow phenomenon has been demonstrated after both renal ischemia and reperfusion (I/R)^[3]^. More recently, Kloner *et al* found that microvascular damage was involved in the genesis of the "no-reflow" phenomenon in dogs^[4]^. In the dogs underwent CAO for 40 min and followed by reperfusion for 90 min, complete CBF restoration occurred after a 40-min ischemia; however, CBF was not completely restored after CAO with a duration of 90 min, confirming that the formation of the no-reflow area was associated with microvascular injury^[4]^. In 1985, Schofer *et al* discovered that the no-reflow phenomenon was observed in patients with ST-segment elevation myocardial infarction (STEMI, *n* = 4)^[5]^. It was found that thrombolysis did not result in the complete CBF restoration. The no-reflow area was documented by scintigraphy with thallium-201 and ^99m^Tc-microalbumin aggregates. All patients had an acute myocardial infarction (AMI), and the onset of chest pain was less than 3 h before hospital admission^[5]^.

In 1989, the vasoconstriction area after successful percutaneous coronary intervention (PCI) was observed in patients (*n* = 5) with STEMI^[6]^. It should be noted that both studies involved small samples of patients^[5–6]^; therefore, these studies cannot be used to assess the incidence of the microvascular obstruction (MVO) phenomenon. It should also be noted that there is no generally accepted definition of the terms "the no-reflow phenomenon" and "MVO". Sometimes these terms are used as interchangeably, with some investigators believing that MVO is one manifestation of the no-reflow phenomenon or the slow flow phenomenon. Currently, investigators use the term "MVO" or the term "the slow flow phenomenon" more often, because the complete no-reflow phenomenon is observed angiographically only in 5% of patients after PCI, and in the remaining patients, there could be a non-complete restoration of CBF^[7]^. Here, the terms "no-reflow" and "MVO" are used synonymously. We will use the term "MVO" more often than the term "no-reflow" in the description of clinical studies, because in our opinion "no-reflow" is a clinical situation where CBF has not been restored in the infarct-related coronary artery. Professor G. Heusch has made a great contribution to the study of MVO^[8]^.

## Incidence, mortality, and prognosis of MVO in patients with AMI

### Incidence

The persistent full no-reflow phenomenon (the Thrombolysis in Myocardial Infarction [TIMI] flow grade 0) can be angiographically found in approximately 5% of patients with AMI and PCI, but MVO may be more common^[7]^. MVO has been diagnosed by the contrast-enhanced magnetic resonance imaging (MRI) in 25% of patients with STEMI^[9]^, diagnosed angiographically in 29% of patients with both STEMI and PCI^[10]^, but detected by MRI in 56% of patients with both STEMI and PCI^[11]^ and diagnosed with transthoracic echocardiography in 50% of patients with both STEMI and PCI^[12]^. MVO also can be detected by the angiographic TIMI flow grade in 25% of patients with STEMI and PCI in one study^[13]^ but in 46% of patients with STEMI and PCI by the same method in another study^[14]^. However, MRI data alone suggested that the incidence of MVO was 37% in patients with STEMI and PCI^[15]^. These data suggest that the incidence of MVO may range between 25% and 56% in patients with STEMI and PCI.

### Mortality

Patients with MVO six months after AMI had a greater number of cardiovascular events than those without MVO^[9]^. The in-hospital mortality rate was approximately 14% in patients with MVO and 3% in those without MVO, with a total mortality of 6% as reported^[16]^. Persistent no-reflow was associated with an increased all-cause mortality during a 3-year follow-up after AMI^[17]^. Patients with STEMI and no-reflow had an increased risk of in-hospital mortality, compared with patients without no-reflow^[14]^.

### Prognosis

MVO predicts a more frequent development of cardiovascular complications in patients with AMI within two years^[9]^. MVO measured by myocardial contrast echocardiography is the most powerful independent predictor of the adverse left ventricular remodeling after STEMI^[18]^. In patients with STEMI undergoing PCI, the no-reflow is a strong independent predictor of 5-year mortality^[10]^. MVO is associated with a larger myocardial infarction, a lower ejection fraction, and a high mortality during five years after AMI^[19]^. MVO is also accompanied by the adverse cardiac remodeling eight months after AMI^[20]^. Major adverse cardiac events during six months after STEMI often occur in patients with MVO detected by MRI^[21]^.

**Table 1:** Microvascular obstruction triggers: analysis of experimental data (continued)

Hypothetical trigger	Species	Experimental model	Effect	Ref.
ROS, Ca^2+^ overload	Rat	Isolated heart	Acetylcholine-induced vasodilation decreased after reperfusion following tBHP administration	[51]
	Rabbit	CAO and reperfusion *in vivo*	Gallopamil and SOD reduced the no-reflow area	[52]
	Dog	CAO and reperfusion *in vivo*	CBF decreased after three hours of reperfusion. Clentiazem reduced the no-reflow zone and reduced infarct size	[54]
	Rabbit	CAO and reperfusion *in vivo*	Verapamil had no effect on the no-reflow area and infarct size	[55]
	Rat	CAO and reperfusion *in vivo*	Verapamil reduced infarct size	[56]
	Pig	CAO and reperfusion *in vivo*	Diltiazem and verapamil reduced the no-reflow area but did not affect infarct size	[57]
	Mouse	CAO and reperfusion *in vivo*	HINT2 overexpression promoted a decrease in the no-reflow area	[58]
	Minipig	CAO and reperfusion *in vivo*	Fosinopril and valsartan reduced the MVO area	[64]
CAO: coronary artery occlusion; CBF: coronary blood flow; CPP: coronary perfusion pressure; ERK1/2: extracellular signal-regulated kinase 1/2; HINT2: histidine triad nucleotide-binding 2; HUVEC: human umbilical vein endothelial cells; LV: left ventricle; MPO: marker of neutrophil myeloperoxidase; MVO: microvascular obstruction; NLRP3: (NOD)-like receptor pyrin domain containing 3; ROS: reactive oxygen species; SOD: superoxide dismutase; tBHP: tert-butyl hydroperoxid.				

## MVO triggers and modulators: an analysis of experimental data

### Endothelial cell injury

In dogs underwent CAO at 20, 40, 60, 90, or180 min intervals, ultrastructural injury of cardiomyocytes was found 20–40 min after the onset of ischemia, but microvascular injury was detected 60–90 min after the onset of ischemia^[22]^. It appears that endothelial cells are more tolerant to ischemia than cardiomyocytes. According to Kloner *et al*, the no-reflow area was distinguished by ultrastructural microvascular damage, including swelling of endothelial cells and the obstruction of the lumen of small vessels^[23]^. The presence of endothelial swelling and peri-capillary edema in animals with no-reflow is beyond doubt^[22]^. However, there is no conclusive evidence of the important role of endothelial swelling in the pathogenesis of MVO, because decongestants have not been used for treatment of MVO. When an isolated rodent heart was subjected to global ischemia for 45 min and reperfusion for 15 min, I/R reduced the lumen of capillaries by approximately 50%, endothelial swelling was developed, and the endothelial cell size was increased by approximately 20%^[24]^. It is unclear whether these alterations induce MVO, even though the antioxidant Trolox increased the lumen of capillaries but did not affect endothelial cell swelling^[24]^.

The glycocalyx is a pericellular matrix layer containing glycoproteins and glycolipids that covers the cell membranes of endothelial cells, epithelial cells, and other cells. I/R induced glycocalyx injury and, as a result, endothelial cell dysfunction^[25]^. I/R injured the endothelial glycocalyx when isolated guinea pig hearts were subjected to global ischemia (15 min) and reperfusion (20 min)^[26]^. However, a correlation analysis between plasma concentration syndecan (a protein of the glycocalyx), thrombomodulin (a protein of endothelial cells), and MVO size had not been performed before (***Table 1***).

**Table 1:** Microvascular obstruction triggers: analysis of experimental data

Hypothetical trigger	Species	Experimental model	Effect	Ref.
Endothelial cell injury	Dog	CAO *in vivo*	Ultrastructural damage of cardiomyocytes, microvascular injury	[22]
	Rat	Isolated heart	Decreased capillary lumen, endothelial edema	[24]
	Guinea pig	Isolated heart	Glycocalyx injury, endothelial cell dysfunction	[25–26]
Microembolization and microthrombi	Dog	CAO and reperfusion *in vivo*	Insignificant decrease in CBF	[27]
	Rabbit	Isolated heart	MVO occurred without thrombosis, platelets and leukocytes	[32]
	Rat	Isolated heart	No-reflow area formation	[33]
	Rat	Isolated heart	CBF decreased in LV outer layers	[34–35]
Leukocyte invasion	Rat	Isolated heart	Decrease in the number of perfused capillaries (approximately 62%)	[81]
	Rabbit	CAO and reperfusion *in vivo*	CY1503 did not reduce infarct size or no-reflow	[82]
	Rat	Isolated heart	Platelets and neutrophils injured coronary arteries in reperfusion	[83]
	Dog	CAO and reperfusion *in vivo*	R15.7 reduced infarct size and the no-reflow area	[53]
	Human	HUVEC, Hypoxia/reoxygenation	Induction of adhesion of neutrophils, ZM-241385 abolished adhesion	[66]
	Rat	CAO and reperfusion *in vivo*	Correlation of MPO with no reflow area	[84]
Platelet aggregation	Rabbit	CAO and reperfusion *in vivo*	Platelets were involved in the pathogenesis of MVO in animals with hypercholesterolemia	[37]
	Minipig	CAO and reperfusion *in vivo*	Tirofiban reduced the MVO area. A Combination of clopidogrel + aspirin had no effect on the MVO area	[39]
	Rat	Isolated heart	An increase in CPP	[40]
	Rat	Isolated heart	Platelets reduced infarct size, activated ERK1/2	[41]
Coronary artery dysfunction	Dog	CAO and reperfusion *in vivo*	Decreased acetylcholine-induced vasodilation. Verapamil reduced impaired vascular relaxation.	[42]
	Dog	CAO and reperfusion *in vivo*	Disruption of endothelium-dependent vasodilation of the infarcted coronary artery	[43]
	Cat	CAO and reperfusion *in vivo*	Decrease in vasodilation caused by acetylcholine. A23187-induced vasodilation was reduced by reperfusion. SOD improved acetylcholine-induced vascular relaxation	[44]
	Pig	Isolated intramyocardial ring of arteries	Reduced vasodilation triggered by bradykinin	[46]
Pericytes	Rat	CAO and reperfusion *in vivo*	I/R blocked blood flow in 40% of capillaries	[47]
	Mouse	CAO and reperfusion *in vivo*	GPR39 knockout reduced the no-reflow area, VC43 also reduced MVO	[48]

### Microembolization and microthrombi

In dogs undergoing CAO (3 h) and reperfusion(3 h), the infarct area of the left ventricle was almost identical between the control (13.4%) and streptokinase-treated groups (13.0%); CBF was decreased by 90% in the subendocardial area at risk (I/R zone) during ischemia; CBF in the subendocardium was 0.33 mL/(min·g) in the control group and 0.38 mL/(min·g) in the streptokinase group during reperfusion; however, these differences were not statistically significant^[27]^. Therefore, thrombosis of coronary microvessels did not play a key role in the pathogenesis of the no-reflow phenomenon. In a later article, Kloner *et al* reported that the aggregation of blood elements could play a greater role in the development of no-reflow^[23]^. Indeed, clinical studies have confirmed the important role of aggregation of platelets^[28–29]^ and erythrocytes in the development of MVO in patients with AMI^[30]^. Professor G. Heusch claimed that he could demonstrate an important role of microembolization and microthrombi in the appearance of no-reflow personal communication^[8]^. However, his opinion is not shared by other investigators.

### MVO without microembolization or microthrombi

In a placebo-controlled trial, it was demonstrated that intracoronary administration of alteplase in patients with STEMI and PIC had no effect on the incidence of MVO^[31]^. In one study, isolated rabbit hearts were subjected to global ischemia (30 or60 min) and reperfusion (90 or 60 min), global ischemia without reperfusion induced edema of the myocardium; the reperfusion triggered an increase in perfusion pressure, indicating the presence of coronary vasospasm and MVO; and coronary artery permeability was measured by recording the passage of ^l25^I-BSA (bovine serum albumin) through the coronary vessels; in addition, vascular permeability increased as the duration of reperfusion increased^[32]^. These findings indicate that MVO can exist without the involvement of thrombosis, platelets, and leukocytes.

In another study, an isolated perfused rodent heart was subjected to global ischemia (60 min) and reperfusion (20 min)^[33]^, and this exposure triggered the formation of a no-reflow area. Therefore, erythrocyte plugging, platelet aggregation, and thrombosis could be excluded as the main causative factors in MVO. When isolated rat hearts were subjected to global ischemia (15, 30, 45, or 60 min) and reperfusion (5 min), the complete restoration of coronary flow (CF) was documented after a 15-min ischemia, and the complete no-reflow area was detected in the subendocardium after a 30-min ischemia with a reduction in CBF by 50% in the middle myocardium; in addition, a decrease in CBF in the subepicardium was detected after a 45-min ischemia^[34]^. These investigators hypothesized that no-reflow could be a result of compressed myocardial contracture. When isolated rat hearts were subjected to global ischemia (30 min) and reperfusion (1, 5, 20, and 60 min), CBF in left ventricle outer layers before ischemia was 13.2 mL/(g·min) but decreased to 4.8 mL/(g·min) after a 1-min reperfusion, and further decreased to 2.5 mL/(g·min) after a 60-min reperfusion^[35]^. These results suggest that the cause of no-reflow is an ischemic injury of coronary arteries, but reperfusion injury also plays a role in the no-reflow phenomenon. In isolated rat heart, global ischemia (30 min) and reperfusion (30 min) resulted in the appearance of no-reflow after I/R of the isolated heart, and that the no-reflow area was occupied 30% of the left ventricle^[36]^.

Therefore, the role of microembolization and microthrombi in the pathogenesis of MVO remains a matter of debate. MVO could develop without the involvement of platelets, leukocytes, and erythrocytes. However, we cannot exclude the involvement of platelets, leukocytes, and erythrocytes in the pathogenesis of MVO *in vivo*.

### Platelet aggregation

To study the role of platelet aggregation in MVO, rabbits underwent CAO (30 min) and reperfusion were used (5.5 h) in the development of MVO^[37]^. It was observed then that the labeled platelet accumulation in the myocardium of hypercholesterolemic rabbits was approximately 4-fold higher than in control animals; the infarct size was also approximately 2-fold higher than in the control group; and the no-reflow area was 4-fold higher than in the control rabbits^[37–38]^. It was also observed that goat anti-rabbit platelet serum reduced the infarct size and the MVO area only in hypercholesterolemic rabbits^[37]^. It appears that platelets are involved in the pathogenesis of MVO in animals with hypercholesterolemia.

Aggregating platelets could disrupt the relaxation of isolated rings of canine coronary arteries. For example, when mini-pigs were subjected to CAO (3 h) and reperfusion (60 min) and also pretreated with the aspirin + P2Y_12_ receptor antagonist clopidogrel or glycoprotein Ⅱb/Ⅲa inhibitor tirofiban for three days before CAO, both clopidogrel and tirofiban significantly reduced platelet aggregation rate; although only tirofiban reduced the MVO area by 71%, a combination of clopidogrel + aspirin had no effect on the MVO area^[39]^. These investigators left this surprising difference between clopidogrel and tirofiban without an explanation.

However, there is evidence that platelets can exhibit cardioprotective properties in I/R of the heart. For example, when isolated rat hearts were subjected to global ischemia (15 min) and reperfusion (10 min), I/R induced disturbances of contractile function and an increase in coronary perfusion pressure (CPP) that indirectly indicated the appearance of MVO; perfusion of hearts with a solution containing rat platelets prevented reperfusion-reduced contractile dysfunction and abolished an increase in CPP, while reperfusion creatine kinase release was also reduced^[40]^.

The cardioprotective effect of platelets was experimentally confirmed by Russo *et al*^[41]^. In his study, the isolated rat hearts were subjected to global ischemia (30 min) and reperfusion (60 min), and platelets of healthy volunteers were added to a perfusion solution prior to global ischemia. In this case, platelets resulted in a decrease in the infarct size by approximately 15%; although platelets of patients with diabetes mellitus did not limit the infarct size, platelets of healthy volunteers induced phosphorylation (activation) of extracellular signal-regulated kinase (ERK1/2); furthermore, platelets of patients with diabetes also had no effect on ERK1/2 phosphorylation, while inhibition of ERK1/2, protein kinase C (PKC), phosphatidylinositol (4,5)-bisphosphate 3-kinase (PI3K) eliminated the cardioprotective effect of platelets^[41]^. It should be noted that ERK1/2, PI3K, and PKC participate in the pre- and postconditioning-induced cardiac tolerance to I/R. It is possible that platelets could release a cardioprotective substance that increases cardiac tolerance to I/R and that platelets could be involved in MVO. It should be noted that the infarct-limiting effect of platelets was weak (−15%). These data were not confirmed by other investigators.

### Coronary artery vasodilation

In dogs underwent CAO (60 min) and reperfusion (60 min), the responses of arterial rings to acetylcholine, an endothelium-dependent vasodilator, were studied; it was found that I/R of the heart reduced acetylcholine-induced vasodilation, whereas the response to nitroprusside, an endothelium-independent vasodilator, was not altered; furthermore, intravenous administration of the L-type Ca^2+^ channel blocker verapamil mitigated the I/R-induced disturbance in vascular relaxation^[42]^. Therefore, it is likely that I/R disturbed endothelium-dependent vasodilation of coronary arteries through Ca^2+^ overload of endothelial cells. However, when dogs were subjected to CAO (2 h) and reperfusion (3 h), and acetylcholine was infused intracoronary in reperfusion, the reperfusion disturbed endothelium-dependent vasodilation of the infarcted coronary artery^[43]^. In another study, cats underwent CAO(90 min) and reperfusion (0, 2.5, 5, 20, 180, or 270 min), and then vasodilation of coronary arterial rings isolated from the left anterior descending (LAD) coronary artery subjected to I/R was studied; in addition, the following compounds were used: acetylcholine, A23187 (an endothelium-dependent vasodilator), and NaNO_2_ (an endothelium-independent vasodilator)^[44]^; the results showed that acetylcholine-induced relaxation of LAD coronary artery rings was not impaired after ischemia without reperfusion, that the reperfusion (2.5 min) reduced acetylcholine-triggered vasodilation by 36%, and that A23187-induced vasodilation decreased after 20-min reperfusion. However, there was no decrease in response to NaNO_2_ in the LAD artery rings in reperfusion; although pretreatment with superoxide dismutase (SOD) improved acetylcholine-induced vascular relaxation in reperfusion, a free radical scavenger N-(2-mercapto propionyl)-glycine did not improve acetylcholine-triggered vasodilation^[44]^. Furthermore, transmission electron microscopy revealed very little endothelial cell damage even after long-term CAO (4.5 and 6.0 h) without reperfusion^[45]^. These results suggest that reperfusion, but not ischemia, is the main cause of I/R injury of coronary arteries in I/R in cats. Therefore, these investigators concluded that reperfusion injured endothelial cells and reduced endothelium-derived relaxing factor release through an increase in superoxide radical production^[44]^.

To study vascular relaxation of intramyocardial artery rings isolated from the ischemic porcine myocardium, Dignan *et al* used bradykinin to stimulate endothelium-dependent vasodilation^[46]^ and sodium nitroprusside, an NO donor, for endothelium-independent vasodilation^[46]^. It was found that ischemia (60 min) disturbed vasodilation, that the vasodilator effect of bradykinin disappeared after90-min of ischemia, and that the vasodilator effect of sodium nitroprusside was not altered by ischemia^[46]^. These results suggest that both reperfusion and ischemia can disrupt endothelium-dependent vasodilation of coronary arteries. It is unclear why the response of coronary arteries to ischemia is so different in cats and pigs.

### The involvement of pericytes in MVO

Pericytes are perivascular cells found in the heart, which are involved in angiogenesis, regulation of CBF, and vascular permeability. Dysfunction of pericytes is also involved in the pathogenesis of MVO. For example, when rats underwent CAO(45 min) and reperfusion (15 min)^[47]^, I/R blocked blood flow in 40% of capillaries in the area at risk; moreover, some evidence was obtained that pericytes could induce vasoconstriction of microvessels in the heart, but adenosine mitigated pericyte constriction and no-reflow^[47]^. Another study showed that the GPR39 receptor was expressed in vascular smooth muscle cells and pericytes, and the activation of this receptor promoted the vasoconstriction of microvessels in myocardial tissue^[48]^. When the wild-type mice and GPR39 knockout mice underwent CAO(45 min) and reperfusion (2 h), the no-reflow area was smaller in the GPR39 knockout mice, compared with the wild-type mice, while the administration of the GPR39, a VC43 receptor antagonist, 30 min before the onset of reperfusion also reduced MVO^[48]^. These investigations suggest that pericytes may be involved in the I/R-induced vasoconstriction of microvessels^[48]^.

### MVO and the adverse post-MI remodeling

In rats underwent CAO (60 min) and reperfusion, scar thickness and the infarct expansion index were assessed four weeks after myocardial infarction in one study, and the results showed that CBF was reduced in the infarcted myocardium, compared with the non-infarcted myocardium; then, a number of perfused capillaries in the infarcted area were found to be closely correlated with the infarct expansion index and significantly correlated with scar thickness, suggesting that MVO may persist for one month after reperfusion and predict adverse myocardial remodeling^[49]^. Another study performed in pigs subjected to CAO (90 min) and reperfusion also showed persistent MVO on days 7–9 after the onset of reperfusion^[50]^. Thus, MVO may be a persistent pathological alteration in CBF, which could promote adverse post-infarction remodeling of the heart.

### Reactive oxygen species and Ca^2+^ overload

What is the trigger of microvascular dysfunction? The aforementioned data suggest that it could be Ca^2+^ overload or oxidative stress^[24,42,44]^. In one study, the isolated perfused rat hearts were subjected to global low-flow ischemia (30 min) and reperfusion (30 min) and the coronary arteries were constricted by the addition of U46619, a stable analogue of thromboxane A2 and the vasodilation were induced by acetylcholine (an endothelium-dependent vasodilator) or an NO donor glyceryl trinitrate (an endothelium-independent vasodilator), and then the measurements were performed before ischemia, after ischemia, or pretreatment with the oxygen radical inducer tert-butyl hydroperoxide (tBHP); the results showed that the vasodilation induced by acetylcholine was reduced by 34% after reperfusion and by 48% after tBHP, while responses to an NO donor were not altered; moreover, tBHP-injured vasodilatation responses to acetylcholine were improved by pretreatment with the L-type Ca^2+^ channel blocker nisoldipine; by contrast, the vasodilator effect of acetylcholine was not improved when the hearts were perfused with L-type Ca^2+^ channel blockers diazepam or verapamil, suggesting that reperfusion or oxidative stress may disturb endothelium-dependent vasodilation^[51]^; however, the investigators provided no explanation as to why nisoldipine could abolish reperfusion or free radical-induced disturbance of vasodilation, but verapamil or diazepam had no effect? In another study, rabbits underwent CAO (30 min) and reperfusion (5.5 h); at the same time, the L-type Ca^2+^ channel blocker gallopamil was administered in CA, and SOD was administered in reperfusion; the results showed that both gallopamil and SOD reduced the infarct size and decreased the no-reflow area by about 60%^[52]^. These data demonstrate that Ca^2+^ overload and oxidative stress are involved in reperfusion injury of coronary arteries. Neutrophils could also be a source of oxygen radicals in injured coronary arteries in reperfusion^[53]^; however, they cannot be a source of oxygen radicals as shown in a study with the isolated rat heart^[54]^, in which dogs underwent CAO (90 min) and reperfusion (6 h), and CBF decreased by 82% in the subendocardium after a 80-min ischemia but completely restored after a 30-min reperfusion, while CBF was reduced by 32% in the subendocardial area at risk after a 3-h reperfusion, compared with the pre-ischemic value, and continued to decline with further reperfusion, but the L-type channel blocker clentiazem given at reperfusion mitigated no-reflow and reduced the infarct size^[54]^.

In one study, rabbits underwent CAO (30 min) and reperfusion (120 min), and verapamil was administered intravenously (5 min prior to reflow) as a bolus of 50 μg/kg and infused at 150 μg/(kg·h) until the end of reperfusion to make the total dose of verapamil 0.35 mg/kg; the results showed that verapamil did not affect the no-reflow area and the infarct size^[55]^. However, our group found that the administration of verapamil at a dose of 0.2 mg/kg5 min before reperfusion reduced the infarct size by 30% in rats with CAO (45 min) and reperfusion(120 min)^[56]^. Therefore, the absence of the infarct-reducing effect of verapamil in Reffelman's study was surprising to us. In another study, when pigs underwent CAO (3 h) and reperfusion (1 h), and then diltiazem and verapamil were injected with intracoronary at a bolus of 2 mg 1 min before reperfusion; the results showed that both diltiazem and verapamil reduced the no-reflow area (*P* < 0.01) but had no effect on the infarct size, suggesting that the L-type channel blockade did not affect the infarct size, if the duration of ischemia was 3 h, but could reduce the no-reflow area within 3 h^[57]^.

Mitochondrial calcium Ca^2+^ overload was also involved in the I/R injury of the heart, and histidine triad nucleotide-binding 2 (HINT2) further regulated the Ca^2+^ level in mitochondria as reported^[58]^, in which HINT2 overexpressed mice and wild-type mice underwent CAO (45 min) and reperfusion; the results showed that HINT2 overexpression promoted a decrease in the no-reflow area, and the same effect was exhibited by Ru360, an inhibitor of Ca^2+^ transport into mitochondria^[58]^. These findings indicate that mitochondrial Ca^2+^ overload is also involved in MVO. Therefore, it appears that reactive oxygen species (ROS) and Ca^2+^ overload may be involved in the pathogenesis of MVO, but surprisingly, some antioxidants and the L-type Ca^2+^ channel blockers cannot prevent the development of MVO.

### Endothelins

One study showed that when the isolated rabbit hearts were subjected to global ischemia (60 min) and reperfusion (60 min), I/R reduced the left ventricular-developed pressure and CPP; but the endothelin receptor antagonist BQ-123 had no effect on reperfusion recovery of contractility and CPP^[59]^. The investogators suggested that endothelins did not play a key role in MVO.

### Na^+^/H^+^ exchanger

One study showed that when rabbits were subjected to CAO (30 min) and reperfusion (180 min) and the Na^+^/H^+^ exchanger inhibitor cariporide (0.3 mg/kg) was also injected intravenously before ischemia, cariporide reduced the infarct size by 62% and promoted a reduction in the no-reflow area by 53%, compared with a decrease in the no-reflow area by 37% with ischemic preconditioning (***Table 2***)^[60]^. Thus, both the inhibition of the Na^+^/H^+^ exchanger and ischemic preconditioning could prevent the appearance of no-reflow, but cariporide exhibited a more pronounced effect, suggesting that the Na^+^/H^+^ exchanger plays an important role in MVO.

**Table 2:** Factors contributing to the attenuation of microvascular obstruction

Hypothetical factor	Species	Experimental model	Effect	Ref.
Na^+^/H^+^ exchanger	Rabbit	CAO and reperfusion *in vivo*	Cariporide reduced infarct size and the no-reflow area	[60]
Nitric oxide	Pig	CAO and reperfusion *in vivo*	NO inhalation resulted in a decrease in infarct size. NO inhalation increased CBF in the area at risk	[61]
Adenosine	Dog	CAO and reperfusion *in vivo*	Adenosine reduced infarct size by decreasing the release of endothelin from the heart during I/R	[65]
	Dog	CAO and reperfusion *in vivo*	Infusion of adenosine during the first hour of reperfusion mitigated MVO	[43]
	Dog	CAO and reperfusion *in vivo*	Adenosine increased CBF in the subendocardium with no change in CBF in the midmyocardium and epicardium	[65]
	Rabbit	CAO and reperfusion *in vivo*	Adenosine had no effect on MVO	[55]
	Minipig	CAO and reperfusion *in vivo*	Adenosine reduced the no-reflow area by 75%	[57]
	Rabbit	CAO and reperfusion *in vivo*	GP531 had no effect on CBF in the area at risk in reperfusion	[67]
	Pig	CAO and reperfusion *in vivo*	It was found that adenosine reduced the no-reflow area by 47%	[68]
Kinases and NO-synthase	Rat	CAO and reperfusion *in vivo*	Tongxinluo reduced the no-reflow area by 80%. Tongxinluo promoted phosphorylation of eNOS and increased PKA activity	[69]
	Minipig	CAO and reperfusion *in vivo*	Simvastatin reduced the no-reflow area by 28%. eNOS is involved in this effect of simvastatin	[71]
K_ATP_ channels	Minipig	CAO and reperfusion *in vivo*	The K_ATP_ channel opener and an NO donor nicorandil reduced infarct size and the no-reflow area	[74–75]
Ischemic conditioning	Rabbit	CAO and reperfusion *in vivo*	Ischemic preconditioning decreased the no-reflow area by 37%	[60]
	Minipig	CAO and reperfusion *in vivo*	Ischemic preconditioning reduced the MVO area by 78%. PKA and eNOS are involved in this effect	[70]
	Minipig	CAO and reperfusion *in vivo*	Remote preconditioning reduced infarct size by 24%, and the no-reflow area by 45%	[76]
	Rabbit	CAO and reperfusion *in vivo*	Post-conditioning had no effect on infarct size and the no-flow	[77]
	Pig	CAO and reperfusion *in vivo*	Ischemic post-conditioning had no effect on infarct size or the MVO area	[78]
	Pig	CAO and reperfusion *in vivo*	Ischemic pre-conditioning reduced infarct size by 50% and the MVO area by 80%. Post-conditioning had no effect on infarct size and the MVO area	[79]
	Pig	CAO and reperfusion *in vivo*	Ischemic pre-conditioning, ischemic post-conditioning, and remote perconditioning decreased myocardial edema and the MVO area	[80]
CAO: coronary artery occlusion; CBF: coronary blood flow; eNOS: endothelial NO-synthase; MVO: microvascular obstruction; NO: nitric oxide; PKA: protein kinase A;				

### Nitric oxide

We have discussed above that NO donors, sodium nitroprusside and NaNO_2_, could induce vasodilation of the coronary arteries even after I/R injury of the heart^[42,44,46]^, which may suggest that vascular muscle cells are more tolerant to I/R than endothelial cells. Besides, in pigs that underwent CAO (50 min) and reperfusion (4 h), the inhalation with NO resulted in a decrease in the infarct size by about 50%, but intravenous administration of an NO donor, nitroglycerin, had no effect; moreover, NO inhalation increased CBF in the area at risk 4 h after the onset of reperfusion, but nitroglycerine did not abolish MVO (***Table 2***)^[61]^. Therefore, a dose of nitroglycerin was not enough for an increase in cardiac tolerance to I/R and prevention of MVO. It appears that NO inhalation only increased the NO level in the myocardial tissue^[61]^.

### β-Adrenergic receptor signaling

In one study, rabbits underwent CAO (30 min) and reperfusion (5.5 h), and the β-adrenergic receptor (β-AR) agonist isoproterenol was then infused [0.1 µg/(kg·min)] during ischemia and reperfusion, the unexplained results showed that isoproterenol increased the infarct size by about 2-fold but had no effect on the no-reflow area^[38]^. In another study, mini-pigs were pretreated for three days with non-selective α_1_- and β-AR blocker carvedilol [1 mg/(kg·day)] or the β_1_- and β_2_-AR antagonist propranolol subjected to CAO (3 h) and reperfusion (1 h); carvedilol was found to reduce the no-reflow area by 70%, but propranolol had no effect^[62]^. It should be noted that carvedilol can not only block the adrenergic receptor but also exhibit antioxidant properties as well as inhibit pyroptosis and stimulate autophagy. Thus, it appears that its protective effect against MVO may be independent of β-AR blockade. Additionally, carvedilol-induced protection of the coronary arteries in I/R was mediated *via* the ATP-sensitive K^+^ channel (K_ATP_ channel) opening and possibly a decrease in the endothelin-1 level in the plasma and myocardial tissue^[62]^. These data suggest that β-adrenergic receptors do not play an important role in MVO.

### Angiotensin Ⅱ

In one study, when the isolated perfused rat hearts were subjected to global ischemia (30 min) and reperfusion (30 min), pretreatment with the angiotensin Ⅱ AT1-receptor antagonist candesartan improved contractility of the heart and reduced the no-reflow area by about 70%^[63]^. Similarly, when mini-pigs underwent CAO (3 h) and reperfusion (2 h), pretreatment with fosinopril (an angiotensin-converting enzyme inhibitor) and valsartan (an angiotensin Ⅱ receptor antagonist) 3 days before CAO could both reduce the MVO area by approximately 30%^[64]^. These findings suggest that angiotensin Ⅱ may be involved in the development of MVO.

### Adenosine

Adenosine decreased the infarct size in CAO and reperfusion by reducing endothelin release from the heart in I/R in dogs^[65]^. It also protected the isolated cardiomyocytes against hypoxia/reoxygenation, protected endothelial cells and coronary arteries against I/R, and prevented adhesion between isolated endothelial cells and neutrophils^[66]^.

In dogs that underwent CAO (2 h) and reperfusion (3 h), intracoronary infusion of adenosine during the first hour of reperfusion mitigated MVO, preventing reperfusion injury of coronary arteries^[43]^. When dogs were subjected to CAO (90 min) and reperfusion(210 min), adenosine infusion in reperfusion(150 min) reduced the infarct size by increasing CBF in the non-ischemic myocardium by about 4-fold, but triggered a small increase in CBF in the subendocardium of the area at risk without alterations in the midmyocardium and epicardium; moreover, adenosine reduced the plasma endothelin-1 level in the blood from the coronary sinus^[65]^. But when rabbits were subjected to CAO (30 min) and reperfusion(120 min) and adenosine was infused intravenously during the reperfusion period, adenosine had no effect on MVO^[55]^. However, when mini-pigs underwent CAO (3 h) and reperfusion (1 h), adenosine infused 30 min before reperfusion and continued to administer during reperfusion could reduce the no-reflow area by 75%^[57]^. Another study also used rabbits that were subjected to CAO (30 min) and reperfusion (3 h), and then GP531 (a pharmacological agent that increases the endogenous adenosine level in myocardial tissue) was infused before, during, and after ischemia; the results showed that GP531 had no effect on CBF in the area at risk in reperfusion (***Table 2***)^[67]^. While, if pigs underwent CAO (45 min) and reperfusion (2 h) with adenosine infused intracoronary at a dose of50 μg/(kg·min) beginning 5 min prior to reperfusion and continued infusion throughout the 2-h reperfusion period, adenosine reduced the no-reflow area by 47%^[68]^.

Therefore, investigations related to the role of adenosine in the prevention of MVO are contradictory. Some investigators have reported that adenosine mitigates MVO^[43,57,68]^, while others have demonstrated that adenosine has no effect on the no-reflow area^[55,65]^. It should be noted that adenosine could increase CBF 4-fold in the non-infarcted myocardium but had virtually no effect on CBF in the area at risk at reperfusion^[65]^. Therefore, it cannot be ruled out that adenosine may induce the appearance of coronary steal, which would exacerbate the course of AMI.

### Kinases and NO-synthase

**Figure 1:**
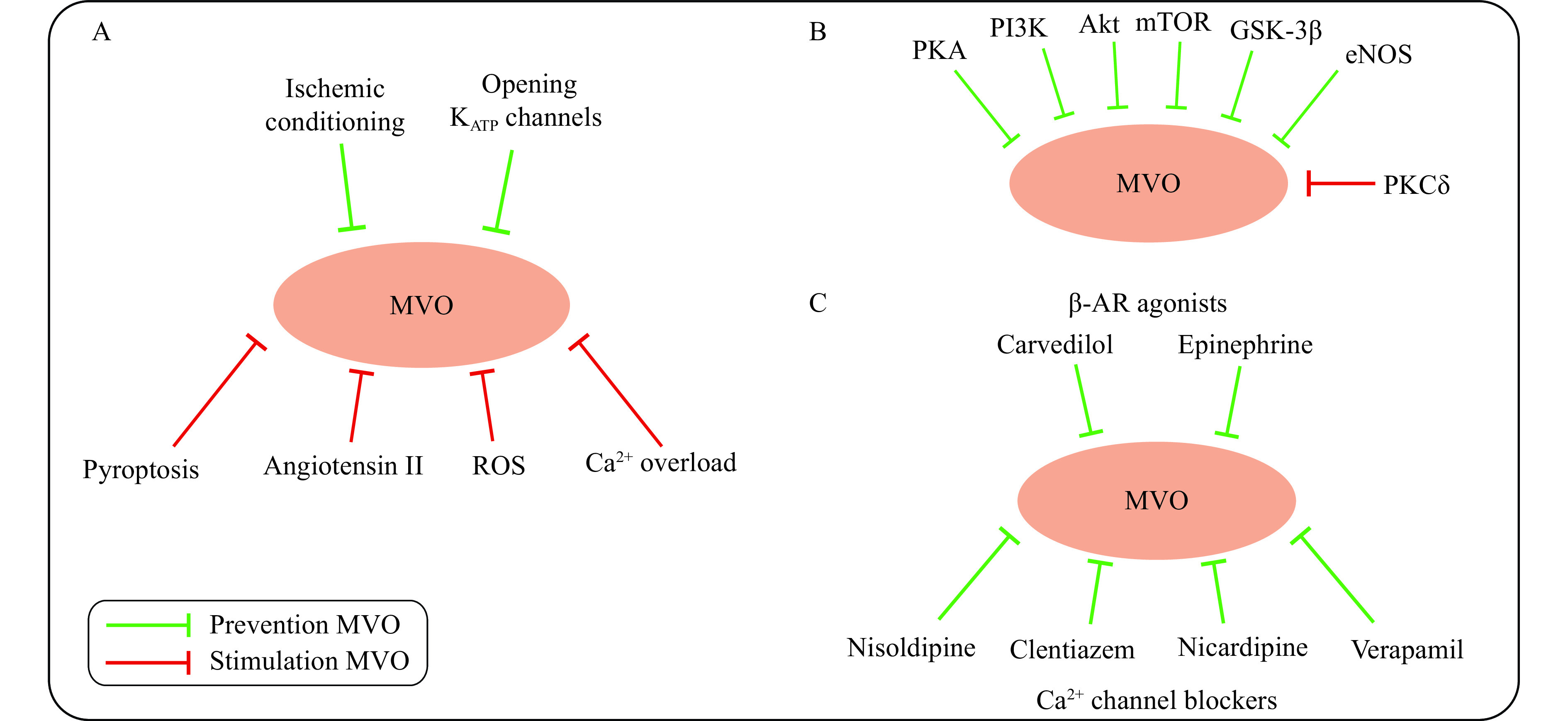
The pathogenesis of microvascular obstruction.

In one study, when rats underwent CAO (90 min) and reperfusion (3 h), Tongxinluo (a traditional Chinese medicine) that was administered intragastrically 1 h before coronary artery occlusion reduced the no-reflow area by 80% (***Table 2***)^[69]^. In mechanism, the inhibitor protein kinase A (PKA) H-89 was demonstrated to be partially abolished, but the NO-synthase (NOS) inhibitor L-NNA completely reversed the reduction in the no-reflow area; also, Tongxinluo could promote phosphorylation of endothelial NOS (eNOS) and increase PKA activity^[69]^; thus, eNOS and PKA may be involved in the protective effect of Tongxinluo. Another study demonstrated that ischemic preconditioning reduced the MVO area by 78% in mini-pigs with I/R of the heart, and that both PKA and eNOS were involved in this protective effect^[70]^. However, when min-pigs underwent a 1.5-h CAO and a 3-h reperfusion, simvastatin (2 mg/kg) that was administered intragastrically 1 h prior to ischemia reduced the no-reflow area by 28%, in which eNOS was involved in the effect of simvastatin^[71]^. Thus, it could be hypothesized that these kinases might be involved in the protection of endothelial cells in I/R of the heart. Besides, it was reported that Tongmai Yangxin, a traditional Chinese medicine, could prevent the appearance of the no-reflow phenomenon in rats through PI3K/Akt/eNOS, cAMP/PKA, and NO/cGMP pathways (***Fig. 1***)^[72–73]^.

Therefore, eNOS, PKA, PI3K, Akt, mTOR, and GSK-3β are probably involved in the protection of coronary arteries against I/R. The role of other kinases, such as PKCε, ERK1/2, AMP-sensitive protein kinase (AMPK), Janus kinase (JAK), epidermal growth factor receptor (EGFR, tyrosine kinase) and Src-kinase (tyrosine kinase) in the protection of coronary arteries against I/R remains unknown, although these kinases are involved in the cardioprotective effect of pre- and post-conditioning.

### K_ATP _channels

K_ATP_ channels have been reported to be involved in fosinopril- and valsartan-induced protection against MVO in mini-pigs with CAO and reperfusion; similarly, simvastatin has also demonstrated a protective effect against MVO in mini-pigs, but this effect can be abolished by 5-hydroxydecanoate, a blocker of mitochondrial K_ATP_ channels (mitoK_ATP_ channels); furthermore, nicorandil, acting as an opener of K_ATP_ channels and an NO donor, has been shown to reduce both the infarct size and the no-reflow area in mini-pigs with I/R, but this effect can be abolished by the NOS inhibitor L-NMMA and the K_ATP_ channel blocker glibenclamide^[74–75]^. Additionally, nicorandil was found to decrease plasma endothelin-1 levels and endothelin-1 content in myocardial tissues, while simvastatin also decreased the area of no-reflow in mini-pigs with I/R, but this effect can be abrogated by glibenclamide^[75]^. Therefore, it is likely that K_ATP_ channels are involved in the vasoprotective effects of carvedilol and adenosine. These data suggest that the opening of K_ATP_ channel may prevent MVO. However, the specific mechanism by which K_ATP_ channel opening protects coronary arteries against I/R, whether through the mitoK_ATP_ channel or the sarcolemmal K_ATP_ channel, remains unclear.

### Ischemic preconditioning

Ischemic preconditioning decreased the no-reflow area by 37% in rabbits with CAO and reperfusion in one study^[60]^. Additionally, ischemic preconditioning reduced the MVO area by 78% in mini-pigs with I/R of the heart in another study^[70]^. The protective effect of preconditioning is mediated thought PKA and eNOS. In mini-pigs underwent CAO (3 h) and reperfusion (2 h), and remote preconditioning was performed with four cycles of ischemia (5 min) and reperfusion (5 min) of lower limb ischemia beginning at 40 min before reperfusion of the heart; the results showed that remote preconditioning reduced the infarct size by 24% and the no-reflow area by 45%, but glibenclamide abrogated both effects of remote preconditioning^[76]^. In rabbits underwent CAO (30 min) and reperfusion (3 h), however, post-conditioning had no effect on the infarct size and the no-flow area^[77]^. Additionally, ischemic post-conditioning in pigs (*n* = 6) also showed no effect on the infarct size or the MVO area^[78]^. Apparently, these findings suggest a potential error in the experimental protocol as a typical manifestation of post-conditioning is a decrease in the infarct size.

In one study involving pigs underwent CAO (40 min) and reperfusion, ischemic preconditioning was found to reduce the infarct size by 50% and the MVO area by 80%; however, post-conditioning had no effect on the infarct size and the MVO area^[79]^. In another study involving pigs subjected to CAO(90 min) and reperfusion (3 h), ischemic preconditioning, ischemic postconditioning, and remote perconditioning all decreased myocardial edema and the MVO area, but only ischemic preconditioning reduced the infarct size^[80]^.

These data indicate that preconditioning and remote perconditioning can decrease the MVO area; however, the molecular mechanism of vasoprotection is unknown. Notably, there is a report suggesting that PKA and eNOS may could a role in the vasoprotective effect of preconditioning^[60]^. Still, it is currently unclear whether remote postconditioning can mitigate the no-reflow phenomenon. Further investigations focusing on the molecular mechanisms of the vasoprotective effect of preconditioning, postconditioning, remote perconditioning, and remote postconditioning will promote the creation of new drugs for treatment of MVO in patients with AMI.

## Inflammation and MVO

### Leukocyte invasion

In one study using the isolated rat hearts, global ischemia (30 min) and reperfusion (15 and 25 min) were induced, followed by three types of Krebs buffer solutions for heart perfusion: (1) a solution containing erythrocytes, (2) a solution containing diluted whole blood (DWB), and (3) a solution containing leukocyte-free DWB^[81]^. The results showed that hearts perfused with a solution containing erythrocytes before ischemia demonstrated a decrease in perfused capillaries (approximately 25%) after 25 min of reperfusion; similarly, hearts perfused with leukocyte-free DWB before ischemia also exhibited a decrease in perfused capillaries (approximately 33%) after reperfusion; furthermore, hearts perfused with DWB containing leukocytes demonstrated a reduction in perfused capillaries (approximately 62%) after reperfusion, and coronary vascular resistance was increased by 76%^[81]^. These findings suggest that both leukocytes and platelets could be involved in reperfusion MVO. However, it should be noted that the no-reflow phenomenon can occur without the involvement of leukocytes and platelets.

I/R induces microvascular injury and triggers the appearance of MVO without leukocytes. For example, in rabbits underwent CAO (30 min) and reperfusion(3 h), ^111^In-neutrophils and ^125^I-BSA were used to measure neutrophil accumulation and microvascular plasma permeability^[81]^. The results showed that I/R resulted in an increase in ^111^In-neutrophils and ^125^I-BSA in the area at risk, that anti-neutrophil serum abolished neutrophil accumulation in myocardial tissue but had no effect on microvascular plasma permeability and that the platelet-activating factor antagonist WEB 2086 (10 mg/kg, intravenously) had no effect on neutrophil invasion and microvascular permeability^[81]^. These data indicate that neither neutrophils nor platelets are involved in I/R-induced microvascular permeability.

In dogs underwent CAO (3 h) and reperfusion (2.5 h), I/R induced neutrophil invasion into the walls of large epicardial coronary arteries; similarly, when dogs subjected to CAO (60 min) and reperfusion (120 min), reperfusion triggered leukocyte invasion in myocardial tissues, and *in vivo* I/R of the dog's heart induced leukocyte plugging in microvessels and no-reflow^[79]^. Additionally, leukocyte depletion decreased manifestations of no-reflow in the dog's heart; furthermore, when dogs underwent CAO (90 min) and reperfusion (2 h), leukocyte depletion promoted a reduction in the infarct size and the no-reflow area^[79]^.

In contrast to the previous findings, one study in rabbits (subjected to CAO for 30 min and reperfusion for 4 h) using CY1503, an inhibitor of selectin adhesion molecules (administered 10 and 25 min after the onset of ischemia), found no significant effect on the infarct size or no-reflow^[82]^. These results cast some doubt on a key role of leukocyte invasion in the pathogenesis of MVO.

**Figure 2:**
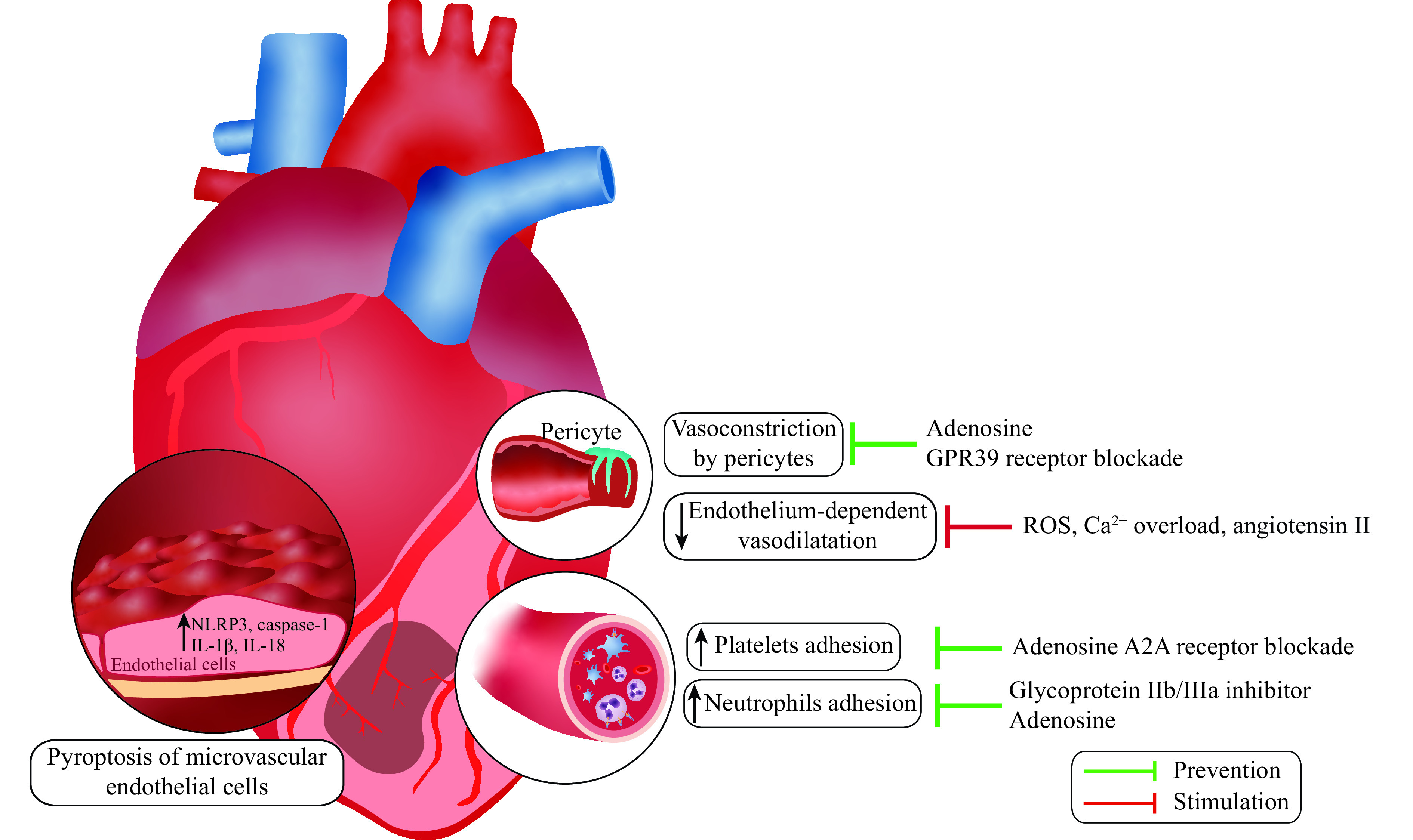
Triggers of microvascular obstruction and contributing factors.

Furthermore, an isolated rat heart model of I/R injury demonstrated that the addition of neutrophils or platelets individually to the perfusion solution resulted in a minor reduction in CF, approximately 5% each; however, when neutrophils and platelets were added to a perfusion solution together, they reduced CF by approximately 50%; furthermore, the selectin-mediated cell adhesion inhibitor sialyl LewisX-oligosaccharide completely abolished the negative effect of co-administration of neutrophils and platelets on CF^[83]^. Based on these findings, it was concluded that platelets and neutrophils injured coronary arteries synergistically in reperfusion through enhancement of platelets' and neutrophils' adhesion in arteries mediated by P-selectin (***Fig. 2***)^[83]^.

In a dog study^[53]^, an exposure to CAO for 90 min followed by reperfusion was performed; it showed that in the control group, regional CBF before ischemia was 80 mL/(100 g·min) and decreased to17 mL/(100 g·min) at the end of ischemia, but, after 10 min of reperfusion, regional CBF was increased to 103 mL/(100 g·min) but then decreased to 60 mL/(100 g·min) after 180 min of reperfusion; in the group treated with the monoclonal antibody R15.7 against the neutrophil CD18 adhesion molecule that was injected 10 min before reperfusion, regional CBF before ischemia was 85 mL/(100 g·min) and decreased to 15 mL/(100 g·min) at the end of ischemia, but, after 10 min of reperfusion, regional CBF was 112 mL/(100 g·min) and decreased to 99 mL/(100 g·min) after 180 min reperfusion^[53]^. In addition, R15.7 antibody decreased the infarct size by approximately 69% and reduced the no-reflow area by approximately 60%^[53]^. These data indicate that neutrophils are involved in late no-reflow.

In a study using human umbilical vein endothelial cells, the cells were subjected to hypoxia (2 h) and reoxygenation (30 min), and subsequently, human neutrophils were added to the culture medium^[66]^. Hypoxia/reoxygenation (H/R) induced adhesion of neutrophils with endothelial cells; however, the adenosine A2A receptor antagonist ZM-241385 abolished adhesion^[66]^. Moreover, in rats underwent CAO (60 min) and reperfusion (2, 4, 6, 12, and 24 h), a marker of neutrophils' myeloperoxidase activity was correlated with the no-reflow area^[84]^. These data indicate that neutrophils are involved in the pathogenesis of MVO in animals with myocardial infarction.

### Pyroptosis and MVO

Pyroptosis is a regulated cell death process that plays an important role in I/R injury of the heart, and nucleotide-binding oligomerization domain (NOD)-like receptor with a pyrin domain 3 (NLRP3) inflammasome plays a key role in pyroptosis^[85]^. There is evidence that pyroptosis is involved in the pathogenesis of MVO as shown in a study^[86]^, in which mice underwent CAO (30 min) and reperfusion (3 or 24 h), reperfusion (3 h) increased NLRP3 expression in myocardial tissue by approximately 2.5-fold, caspase-1 activity increased by 2-fold, the interleukin-1β (IL-1β) level increased by approximately 10-fold, IL-18 content increased by 10-fold; moreover, intraperitoneal administration of inflammasome inhibitor BAY 11-7028 reduced macrophage and neutrophil invasion in myocardial tissue and decreased the infarct size^[86]^. H/R might induce NLRP3 inflammasome and caspase-1 expression in cardiac microvascular endothelial cells (CMECs), increase IL-1β and IL-18 levels in CMECs, and trigger lactate dehydrogenase release, while H/R had no effect on NLRP3 inflammasome expression and IL-1β content in isolated cardiomyocytes^[86]^. It appeared that H/R induced pyroptosis of CMECs and that CMECs were more sensitive to the H/R-induced pyroptosis than cardiomyocytes.

## Triggers and modulators of MVO: analysis of clinical data

### Microembolization and microthrombi

According to Kloner and Heusch, the therapy for no-reflow may be an important approach to improving the outcome for patients with AMI and PCI^[8,23]^. In one study including 78 patients with STEMI and PCI, blood samples were drawn from the aorta and the coronary artery for microparticle detection; it was observed then that microparticle content in the coronary artery was associated with MVO, as evaluated by myocardial blush grade (MBG)^[87]^ (***Table 3***). Based on these findings, the investigators suggested that microparticles could be involved in MVO^[87]^. However, these data have not been consistently confirmed by other investigators; therefore, the key role of microembolization and microthrombi in MVO is questionable.

**Table 3:** Microvascular obstruction triggers: analysis of clinical data

Hypothetical trigger	Disease	Effect	Ref.
Microembolization and microthrombi	STEMI + PCI	The content of microparticles in the coronary artery was associated with MVO	[87]
Platelet aggregation	STEMI + PCI	Correlations were found between the frequency of MVO and ADP-induced platelet aggregation/ aggregation of neutrophil platelets/aggregation of monocyte platelets	[88]
	STEMI + PCI	MVO was more frequently observed in the high platelet reactivity group	[90]
Disturbance of blood viscosity	STEMI/NSTEMI/SA + PCI	Whole blood viscosity was higher in patients with MVO	[30]
Inflammation	STEMI + PCI	Microvascular obstruction score positively correlated with the CRP level and leukocytes. The CRP level was a predictor of MVO in patients with STEMI. The high interleukin-6 level was a predictor of MVO. Patients with MVO had a higher serum level of interleukin-18	[12,30,96–97]
Ca^2+^ overload	STEMI + PCI	The L-type Ca^2+^ channel blockers induced endothelium-independent vasodilation of coronary arteries, Verapamil alleviated MVO in patients with STEMI	[98–99]
	STEMI + PCI	Plasma endothelin-1 on admission was accompanied by MVO and increased long-term mortality	[100]
Diabetes	AMI + diabetes	Hyperglycemia was associated with MVO	[110]
AMI: acute myocardial infarction; CBF: coronary blood flow; CRP: C-reactive protein; MVO: microvascular obstruction; NSTEMI: non-ST-elevation myocardial infarction; NPY: neuropeptide Y; PCI: percutaneous coronary intervention; SA: stable angina; STEMI: ST-elevation myocardial infarction; TIMI: thrombolysis in myocardial infarction.			

### Platelet aggregation

In a study involving patients with STEMI and PCI, MVO was detected using MRI^[88]^. The results showed that MVO scores for no-reflow were lower in patients who received aspirin than in patients who did not receive aspirin; when MVO was evaluated by MRI in patients with STEMI and PCI, the incidence of MVO was significantly correlated with ADP-induced platelet aggregation, platelet-neutrophil aggregation, and platelet-monocyte aggregation^[88]^, suggesting that platelets may play an important role in MVO. Thromboxane A2, synthesized and released by platelets, can induce vasoconstriction, while aspirin may inhibit platelet cyclooxygenase-1 activity that is required for thromboxane A2 synthesis; however, chronic administration of aspirin can trigger a reduction in the serum level of thromboxane B2, a stable metabolite of thromboxane A2^[89]^. These data indirectly suggest that thromboxane A2 could be involved in MVO. Moreover, in another study including patients with STEMI and PCI, MVO was more frequently observed in the high platelet reactivity group than in the low platelet reactivity group^[90]^, also indicating an important role of platelets in MVO. However, convincing data on the involvement of thromboxane in no-reflow are not yet available.

### Coronary artery vasodilation

Currently, there is no clarity about the role of disturbance of endothelial dependent vasodilation in MVO, because acetylcholine, an endothelial-dependent vasodilator, has not been used for the treatment of MVO in patients with AMI. Other pharmacological agents used for treatment of MVO in patients with AMI can induce either endothelium-independent vasodilation (L-type Ca^2+^ channel blockers, NO donors, and nicorandil) or both endothelium independent and dependent relaxation of coronary arteries (β-AR agonists and adenosine)^[91]^.

### Disturbance of blood viscosity

It has been reported that acute coronary syndrome is accompanied by an increase in whole blood viscosity^[92]^. In contrast, Fracassi *et al* detected that whole blood viscosity was higher in patients with STEMI + MVO than in patients with STEMI alone (*P* < 0.001)^[30]^. Therefore, whole blood viscosity could be involved in no-reflow in patients with AMI, although further research is required.

### MVO and adverse post-MI remodeling

It was reported that patients with AMI + MVO showed a progressively increase in left ventricular volumes, compared with patients without MVO six months after AMI^[93]^. In addition, there was a positive correlation among severe MVO, infarct size, and adverse myocardial remodeling six months after AMI^[94]^. Similar findings have been reported by other investigators, supporting that MVO can predict adverse post-infarction myocardial remodeling. However, it should be noted that some investigators could not find the association between MVO and post-infarction remodeling of the heart.

### The involvement of inflammation

The MOV score was positively correlated with both plasma C-reactive protein (CRP) levels and leukocytes in STEMI patients treated by PCI, while the peak CD14^+^CD16^−^ monocyte level was higher in STEMI patients with MVO than in STEMI patients without MVO^[95]^. Both CRP and interleukin-6 levels were reported to be predictors of MVO in STEMI patients underwent PCI^[96]^. STEMI patients with MVO had a higher serum level of interleukin-18 than those without MVO^[97]^. Therefore, CRP and interleukins may be involved in MVO formation.

### Reactive oxygen species and Ca^2+^ overload

The role of ROS in the pathogenesis of no-reflow has not been studied before in AMI patients. However, the L-type Ca^2+^ channel blockers induced endothelium-independent vasodilation of coronary arteries^[98]^, while verapamil could alleviate MVO in patients with STEMI^[99]^. Thus, it could be hypothesized that the Ca^2+^ overload of vascular smooth muscles might be involved in MVO.

### Endothelins, neuropeptide Y, vasopressin

Endothelin-1 is a potent vasoconstrictor peptide. In STEMI patients (*n* = 128) underwent PCI, the plasma endothelin-1 level on admission was associated with both MVO (assessed by MRI) and an increased long-term mortality^[100]^, which suggests that an increase in the endothelin-1 level may promote MVO. Neuropeptide Y (NPY) is another potent vasoconstrictor released from sympathetic endings together with norepinephrine, and intracoronary infusion of NPY induced coronary vasospasm in humans. For example, STEMI patients with angiographic no-reflow had a higher plasma NPY level than those without angiographic no-reflow^[101]^. Other investigators found no differences in the TIMI flow scores between STEMI patients with a low or high NPY level in coronary sinus, although the index of microcirculatory resistance was higher in patients with a high NPY concentration in coronary sinus^[102]^. Arginine vasopressin could induce coronary microvessel spasm and ST elevation in rats^[103]^ as well as contractile response of coronary arterioles in humans^[104]^; however, its role in MVO was not studied before. These findings indicate that endothelin-1 and NPY may participate in MVO. It is too early to draw a final conclusion about the role of NPY and endothelin-1, because correlation analysis between endothelin-1 and NPY levels and the no-reflow area has not been performed.

### Nitric oxide

The TIMI flow grade was improved in AMI patients with PCI treated by intracoronary administration of sodium nitroprusside^[105]^. However, in a later study with a relatively larger patient group, intracoronary administration of sodium nitroprusside did not improve the coronary blood flow in AMI patient underwent PCI^[106]^. These findings suggest that nitroprusside, an NO donor, can not mitigate MVO. It is possible that other NO donors will be more effective.

### β-Adrenergic receptors

There is a study, in which intracoronary administration of epinephrine was reported to completely reversed no-reflow in 9 of 12 STEMI patients treated with PCI^[107]^. It should be noted that the patient sample in this study is very small; therefore, it is unclear whether β-AR agonists could alleviate MVO.

### Adenosine

Intracoronary administration of adenosine reduced the incidence of MVO in patients with AMI treated by PCI^[108]^, which suggests that adenosine may alleviate MVO.

### K_ATP _channels

Nicorandil is not only an NO donor but also a K_ATP_ channel opener. Pinacidil, a K_ATP_ channel opener, triggers endothelium-dependent vasodilation of coronary arteries, while nicorandil as an NO donor resulted in endothelium-independent vasodilation of coronary arteries. Nicorandil reduced the incidence of MVO by more than 50% in AMI patients underwent coronary angioplasty^[109]^; however, sodium nitroprusside, another NO donor, did not prevent no-reflow^[106]^. Thus, it can be hypothesized that nicorandil may trigger vasodilation through NO and K_ATP_ channel opening.

### Diabetes

It was reported that hyperglycemia was associated with MVO in patients with AMI and diabetes^[110]^. However, there were data suggesting that the MVO size was identical in patients with and without diabetes^[79]^. These investigators did not analyze the interaction between MVO, insulin-dependent diabetes, and type 2 diabetes. Therefore, the mechanism of diabetes-induced aggravation of MVO remains unclear.

## Intra-myocardial hemorrhage

MVO is often associated with intra-myocardial hemorrhage (IMH). For example, a combination of MVO and IMH was observed in 41%–54% of STEMI patients underwent PCI, and the hemorrhage area was approximately 3% of the left ventricular mass^[111]^. Fernández-Jiménez *et al* found that the IMH area reached a maximum after 24 h of reperfusion and was approximately 4% in pigs with CAO (40 min) and reperfusion (24 h), while the maximum MVO area was observed in these pigs after 120 min reperfusion^[79]^. Additionally, the maximum IMH area was detected in rats with a 90-min CAO after 48-h reperfusion^[112]^.

MVO preceded microvascular destruction and IMH, and the appearance of IMH was correlated with the adverse ventricular remodelling and worse outcome in AMI patients, while the large IMH area was associated with a late reperfusion and the duration of ischemia^[113]^. IMH was developed after CAO lasting 40–120 min and reperfusion in pigs^[79]^; however IMH did not occur in the infarcted myocardium unless successful reperfusion, and the IMH area was correlated with the MVO area and the infarct size^[114]^. IMH often develops in STEMI patients who have deeper and wider Q waves, while antiplatelet and anticoagulant therapy could promote the appearance of IMH in AMI patients who received PCI^[115]^.

The pathogenesis of IMH remains poorly understood. Classic postconditioning did not prevent IMH or decreased the infarct size in pigs with CAO (40 min) and reperfusion (24 h), while classic preconditioning could reduce the IMH area and the infarct size in these pigs^[79]^.

## Treatment for microvascular obstruction

In a study of STEMI patients underwent PCI, who received aspirin incombination with third-generation P2Y_12_ antagonists (prasugrel or ticagrelor) or the second-generation P2Y_12_ antagonist clopidogrel in addition to aspirin, the incidence of MVO as assessed by MRI was 66% in patients who received clopidogrel, and 49% in patients who received prasugrel or ticagrelor^[116]^. In another study of STEMI patients who received aspirin and clopidogrel orally before PCI as well as received glycoprotein Ⅱb/Ⅲa inhibitor tirofiban intravenously or intracoronary, the incidence of MVO as evaluated by MRI was reduced in patients with intracoronary tirofiban administration, compared with those patients with intravenous administration of tirofiban^[28]^.

Can adenosine reduce the incidence of MVO? As Naghshtabrizi *et al* reported, intracoronary administration of adenosinehave could reduce the incidence of MVO in AMI patients and PCI^[108]^. However, Niccoli *et al* found that intracoronary administration of adenosine had no effect on the incidence of MVO in STEMI patients (*n* = 160) underwent PCI^[117]^. Similar data were also obtained by Nazir *et al* in another study that included patients (*n* = 168) with STEMI and PCI^[106]^. The last two studies are more credible, because they included a larger number of patients. However, it should be noted that adenosine could induce coronary steal and aggravate ischemic/reperfusion injury of the heart in patients with AMI^[118]^. Thus, the use of adenosine for the treatment of AMI and MVO is not recommended.

Can NO donors and K_ATP_ channel openers prevent MVO? As mentioned above, sodium nitroprusside had no effect on the MVO area, while nicorandil could reduce the incidence of MVO by 50% in AMI patients (*n* = 81) with coronary angioplasty^[109]^. Other studies found that an combined intracoronary administration of nicorandil and adenosine could decrease the incidence of no-reflow by 40%^[119]^, and intracoronary infusion of nicorandil alone could also reduce the incidence of MVO in STEMI patients (*n* = 170) underwent PCI^[120]^.

Non-toxic doses of β-AR agonists could increase CBF without hypoxia in dogs with intact coronary arteries^[121]^. However, intracoronary norepinephrine infusion increased myocardial O_2_ consumption and induced myocardial hypoxia in the presence of coronary stenosis^[122]^, and intravenous infusion of a non-toxic dose of isoproterenol induced an increase in the infarct size in rabbits^[38]^. Therefore, the use of β-AR agonists in AMI patients could aggravate I/R injury of the heart. However, these data did not preclude a clinical study of the efficacy of intracoronary administration of epinephrine for treatment of no-reflow (TIMI 0–1, MBG 0–1) in PCI in patients with AMI, which found that epinephrine could significantly improve CBF after PCI in these patients, compared with patients who did not receive epinephrine^[123]^.

L-type Ca^2+^ channel blockers could induce vasodilation of coronary arteries. Intracoronary injection of verapamil reduced the MVO area in AMI patients with PCI^[99]^. Intracoronary infusion of nicardipine could also alleviated no-reflow in 71 of 72 AMI patients underwent PCI^[124]^. Moreover, co-administration of nicardipine, adenosine, and nitroglycerine was reported to reverse the no-reflow in AMI patients with PCI^[125]^. Thus, L-type Ca^2+^ channel blockers may be used to prevent and reverse MVO. Data on treatment are presented in ***Table 4***.

**Table 4:** Treatment for microvascular obstruction

Disease	Compound	Effect	Ref.
STEMI + PCI	Prasugrel/ticagrel + aspirin or clopidogrel + aspirin	Prasugrel/ticagrelor were associated with smaller infarct size and lower MVO incidence versus clopidogrel	[116]
STEMI + PCI	Tirofiban + aspirin and clopidogrel	Reduction in the incidence of MVO	[28]
STEMI + PCI	Adenosine	Had no effect on the incidence of MVO	[106–117]
AMI + PCI	Nicorandil	Reduction the incidence of MVO by 50%	[109]
AMI + PCI	Nicorandil + adenosine	Reduction the incidence of no-reflow by 40%	[119]
STEMI + PCI	Nicorandil	Reduction the incidence of MVO	[120]
AMI + PCI	Epinephrine	Significant improvement CBF	[123]
AMI + PCI	Verapamil	Reduction the MVO area	[99]
AMI + PCI	Nicardipine	Reduction no-reflow in 71 of 72 patients	[124]
AMI + PCI	Nicardipine + adenosine + nitroglycerine	Reversion no-reflow	[125]
AMI: acute myocardial infarction; CBF: coronary blood flow; MVO, microvascular obstruction; PCI: percutaneous coronary intervention; STEMI: ST-elevation myocardial infarction.			

## Conclusions

There is no convincing animal evidence that microembolization and microthrombi are involved in no-reflow with CAO and reperfusion. Neutrophils and platelets are suggested to be involved in the pathogenesis of MVO in animals with CAO. No-reflow was observed in studies performed with isolated perfused hearts subjected to I/R^[34]^. Therefore, it could be hypothesized that I/R-induced injury of coronary arteries may play a key role in the pathogenesis of MVO.

No-reflow was found to be associated with disturbances of coronary artery vasodilation in animals with MI^[42-44]^. There is also evidence that pericytes could be involved in I/R-induced vasoconstriction of microvessels, and angiotensin Ⅱ could be involved in the appearance of MVO, while MVO may participate in the adverse post-infarction myocardial remodeling. Pyroptosis may participate in reperfusion microvascular endothelial injury, while eNOS, PKA, PI3K, Akt, mTOR, and GSK-3β are probably involved in the protection of coronary arteries against I/R. The K_ATP_ channel opening may prevent MVO, and ischemic preconditioning and remote perconditioning may also reduce the MVO area.

There is clinical evidence that platelets could be involved in no-reflow in AMI patients. There is an indirect evidence that Ca^2+^ overload of vascular smooth muscle cells was involved in no-reflow in AMI patients^[125]^. There is also an indirect evidence that inflammation could participate in no-reflow. It is possible that an increased blood viscosity promotes MVO. Adenosine and sodium nitroprusside may not be able to prevent the appearance of MVO, but nicorandil can be used for therapy of MVO. P2Y_12_ antagonists and glycoprotein Ⅱb/Ⅲa inhibitor tirofiban were low effective in treatment for no-reflow, and L-type Ca^2+^ channel blockers remain the most effective drugs for treatment of no-reflow. Although epinephrine can mitigate MVO in patients with AMI and PCI, questions remain.

There is no direct evidence that MVO is associated with injury of endothelial cells in patients with AMI and in animals with CAO, or some indisputable evidence of the involvement of inflammation in MVO in animals with CAO and in patients with AMI. The role of necroptosis, apoptosis, autophagy, and ferroptosis in reperfusion microvascular endothelial injury has also not been studied. It is unclear whether remote postconditioning or adaptation to hypoxia could prevent MVO. The role of PKCε, ERK1/2, AMPK, JAK, EGFR, and Src-kinase in the protection of coronary arteries against I/R remains unknown. The role of thromboxane A, vasopressin, NPY, and ROS in MVO was also not studied before.

Finally, the possibility of antagonists of thromboxane A2 receptor, angiotensin Ⅱ receptors, and NPY antagonists to prevent MVO in patients with AMI and animals with CAO has not been studied, and the efficacy of cardioprotective peptides (opioid peptides, apelins, urocortin-2, and adrenomedullin) for treatment of no-reflow has not been studied before.
